# Complementary Embryonic and Adult Cell Populations Enhance Myocardial Repair in Rat Myocardial Injury Model

**DOI:** 10.1155/2019/3945850

**Published:** 2019-11-03

**Authors:** Sergio Li Calzi, Todd Cook, Domenico G. Della Rocca, Juan Zhang, Vinayak Shenoy, Yuanqing Yan, Andrew Espejo, Anandharajan Rathinasabapathy, Max H. Jacobsen, Tatiana Salazar, George E. Sandusky, Lynn C. Shaw, Keith March, Mohan K. Raizada, Carl J. Pepine, Michael J. Katovich, Maria B. Grant

**Affiliations:** ^1^Department of Ophthalmology and Visual Sciences, University of Alabama at Birmingham, Birmingham, AL 35294-0001, USA; ^2^Department of Medicine, IUPUI, Indianapolis, IN 46202, USA; ^3^Department of Medicine, University of Florida, Gainesville, FL 32611, USA; ^4^Department of Pharmacodynamics, University of Florida, Gainesville, FL 32611, USA; ^5^Department of Physiology and Functional Genomics, University of Florida, Gainesville, FL 32611, USA; ^6^Department of Biostatistics, University of Texas MD Anderson Cancer Center, Houston, TX 77030, USA; ^7^Allergy, Pulmonary, and Critical Care Medicine, Vanderbilt University Medical Center, Nashville, TN, USA; ^8^Pathology and Laboratory Med., IUPUI, Indianapolis, IN 46202, USA

## Abstract

We compared the functional outcome of Isl-1^+^ cardiac progenitors, CD90^+^ bone marrow-derived progenitor cells, and the combination of the two in a rat myocardial infarction (MI) model. Isl-1^+^ cells were isolated from embryonic day 12.5 (E12.5) rat hearts and expanded in vitro. Thy-1^+^/CD90^+^ cells were isolated from the bone marrow of adult Sprague-Dawley rats by immunomagnetic cell sorting. Six-week-old female Sprague-Dawley rats underwent permanent left anterior descending (LAD) coronary artery ligation and received intramyocardial injection of either saline, Isl-1^+^ cells, CD90^+^ cells, or a combination of Isl-1^+^ and CD90^+^ cells, at the time of infarction. Cells were delivered transepicardially to the peri-infarct zone. Left ventricular function was assessed by transthoracic echocardiography at 1- and 4-week post-MI and by Millar catheterization (-dP/dt and +dP/dt) at 4-week post-MI. Fluorescence *in situ* hybridization (Isl-1^+^cells) and monochrystalline iron oxide nanoparticles labeling (MION; CD90^+^ cells) were performed to assess biodistribution of transplanted cells. Only the combination of cells demonstrated a significant improvement of cardiac function as assessed by anterior wall contractility, dP/dt (max), and dP/dt (min), compared to Isl-1^+^ or CD90^+^ cell monotherapies. In the combination cell group, viable cells were detected at week 4 when anterior wall motion was completely restored. In conclusion, the combination of Isl-1^+^ cardiac progenitors and adult bone marrow-derived CD90^+^ cells shows prolonged and robust myocardial tissue repair and provides support for the use of complementary cell populations to enhance myocardial repair.

## 1. Introduction

Despite recent advances in medical therapy, ischemic heart disease remains one of the leading causes of morbidity and mortality worldwide. Since MI results in irreversible damage to the left ventricular wall leading to remodeling and dysfunction, development of treatments is aimed at repairing the muscular tissue and vascular network is now considered a major therapeutic challenge.

The optimal stem cell type for regenerating the heart has been under debate for many years. The adult heart contains its own reservoir of endogenous cardiac stem cells that can, to some extent, generate new cardiomyocytes [1, 2]. Cardiac precursors have been generated from c-kit^+^ [1–4], Sca-1^+^ [5, 6], side population (SP) cells expressing Abcg2 [7], and first and second heart field cells (Tbx5^+^ and LIM homeodomain transcription factor Islet-1 (Isl-1), respectively) [2, 8–11]. Isl1 was found to distinguish this key stem cell population derived from the second heart field [8].

Isl1^+^ progenitor cells have been shown to migrate into the developing heart, giving rise to the outflow tract, the majority of the cells in the right ventricle and the atria, and a portion of cells in the left ventricle [8]. *In vivo* cell lineage tracing in mouse embryos using the Cre-loxP strategy has confirmed that Isl1^+^ progenitors contribute to more than two-thirds of the cells in the embryonic heart [11–13]. Taken together, these studies provide evidence that Isl1^+^ progenitors represent true cardiovascular precursors giving rise to cardiac muscle, parts of the conduction system, and endothelial/smooth muscle cells throughout the proximal aorta, pulmonary trunk, and the branches of the proximal left and right coronary arteries. Previously, it was reported that Isl1 expression was downregulated as soon as the Isl1^+^ cells enter the heart [8, 10]. However, recently evidence of their presence in the adult heart [10] has been demonstrated suggesting that this population could be utilized for allogeneic and perhaps even autologous stem cell therapy.

CD90^+^ cells represent a subfraction [14] of mesenchymal stem cells (MSCs). MSCs, mediators of immune modulation and suppression, are able to improve transplant engraftment, treat graft-versus-host disease, and suppress T cell responses and have shown great therapeutic potential [15]. Their immune modulatory capacity is mediated through cell-to-cell contact and cytokine secretion. Exogenous CD90^+^ cells have also been shown to enhance vascular repair paracrine regulators of blood vessel growth. MSCs have been widely used for cardiac tissue repair either alone [16–24] or in combination with adult c-kit^+^ cardiac progenitor cells (CPCs) [25, 26].

With the advent, subsequent wide spread utilization of iPSC technology and now initiation of clinical trials utilizing these cells, the possibility of generating large numbers of cells with fetal phenotypes and surface markers is now feasible. Embracing this notion of the future feasibility of utilizing cells with a fetal phenotype, we tested the efficacy of Isl1^+^ progenitor cells in myocardial repair following MI in a rat model. We utilize these cells alone and in combination with CD90^+^ cells postulating that because these unique populations target different pathogenic mechanisms/pathways, they would be more effective in combination. CD90^+^ would serve to modulate local inflammation at sites of myocardial injury, while exogenous cardiac Isl-1^+^ stem cells would foster direct cardiomyocyte repair.

## 2. Materials and Methods

### 2.1. Cardiac Progenitor Cell Isolation

Isl1^+^ cells were isolated from rat fetal hearts by differential plating by adaptation of a mouse cell isolation protocol [11] and expanded in culture. Briefly, the hearts from embryonic day 12.5 (ED12.5) rats were cut into four pieces, washed repeatedly in ice-cold Hank's balanced salt solution (HBSS) without Ca^2+^, and predigested overnight in 0.5 mg/ml trypsin in HBSS at 4°C, under constant shaking, to remove blood and dead cells. Cardiac cells were obtained by four rounds of 10 min digestions with 240 U/ml collagenase type II (Worthington, Lakewood, NJ) in HBSS at 37°C. The mesenchymal cell fraction containing most Isl-1^+^ progenitor cells was separated from myocytes and endothelial cells by two rounds of 1-hour differential plating on plastic in DMEM/M199 (4 : 1 ratio) medium containing 10% horse serum and 5% fetal bovine serum at 37°C in the presence of 5% CO_2_. Under these conditions, preferentially mesenchymal and precursor cells attach on plastic. After rigorous washing to remove nonadherent cells, the remaining cardiac mesenchymal cells (CMC) and cardioblasts were cultured for 48 h in DMEM containing penicillin (100 U/ml), streptomycin (100 *μ*g/ml), HEPES (25 mM), glutamine (2 mM), 10% newborn calf serum, and 5% fetal bovine serum (FBS). On the second day in culture, medium was changed to DMEM/F12 containing B27 supplement (StemCell Technologies, Inc.) and 10% FBS to stimulate Isl-1^+^ progenitor growth.

### 2.2. CD90^+^ Cell Isolation

Bone marrow preparations from tibiae and femurs of adult male Sprague-Dawley rats (Charles River Laboratories International, Inc., Wilmington, MA) were obtained aseptically, and after a series of washes and centrifugations, a Thy-1^+^-enriched cell suspension was obtained by immunomagnetic cell selection using a customized kit (StemCell Technologies, Inc., Vancouver, Canada) as per the manufacturer's protocol.

Briefly, whole bone marrow was flushed out of the bones with ice-cold PBS using a 27G needle. The mixture was then centrifuged at 340 g for 10 minutes and the supernatant discarded while the pellet was suspended in 2% FBS and 1 mM EDTA PBS. The resulting cell suspension underwent a negative selection step in which CD4^+^, CD5^+^, CD8a^+^, and OX43^+^ cells were discarded followed by a positive selection step in which the cell suspension was enriched for CD90^+^/Thy-1^+^ cells using a phycoerythrin- (PE-) conjugated mouse anti-rat CD90/Thy-1 antibody (Abcam, Cambridge, MA) and a PE selection kit (StemCell Technologies, Inc.).

### 2.3. Monochrystalline Iron Oxide Nanoparticle (MION) Labeling

For cell tracking purposes, Isl-1^+^ cells were labeled with monochrystalline iron oxide nanoparticles (MION) as previously described [27]. Briefly, confluent cultures of Isl-1^+^ cells were incubated in 10% fetal bovine serum-supplemented medium with 20 *μ*M (1 : 500 dilution) of MIONs in 5% CO_2_ at 37°C overnight. After incubation, cells were washed two times in PBS before trypsinization in order to remove the noninternalized MIONs.

An aliquot of Isl-1^+^ cells was seeded on chamber slides and used for Prussian Blue staining to evaluate MION cell uptake.

### 2.4. Reverse Transcription-Polymerase Chain Reaction

Cell extract total mRNA was isolated (RNeasy kit; Qiagen Inc.) as per the manufacturer's instructions. The mRNA was then transcribed (iScript™ cDNA Synthesis Kit; Bio-Rad, Hercules, CA), and real-time PCR was performed (ABI Master Mix; ABI Biosystems, Foster City, CA). Primers for rat ISL-1, Nkx2.5, and GATA-4 were purchased from ABI Biosystems. All samples were normalized to beta actin (ABI Biosystems) for 60 cycles and all reactions were performed in triplicate.

### 2.5. Immunofluorescence

To assess the purity of the cells, we performed immunofluorescence studies. All the steps, unless otherwise indicated, were performed at room temperature. Cultured cells were fixed in 4% buffered paraformaldehyde (PFA) for 10 minutes and then washed in PBS three times. After a common permeabilization step in 0.2% Triton X-100 in PBS for 10 minutes and an additional three washes in PBS, one set of samples was treated as follows: nonspecific binding was blocked by incubation in 10% normal goat serum for 1 hour and then incubated overnight at 4°C in rabbit anti-Islet-1 (Abcam, Cat. # ab20670, 2 *μ*g/ml) or rabbit anti-GATA-4 (Abcam, Cat. # ab61170, dil. 1 : 500). After three additional washes in PBS, samples were incubated for 1 hour in FITC-conjugated goat anti-rabbit IgG antibody (Vector Laboratories, Burlingame, CA, Cat. # FI-1000, 10 *μ*g/ml). Another set of samples was blocked in 10% normal rabbit serum for 1 hour and then incubated in goat anti-Nkx2.5 (Santa Cruz, Cat. # sc-8697, dil. 1 : 50) overnight at 4°C. Then, samples were incubated in AlexaFluor® 488-conjugated rabbit anti-goat antibody (Invitrogen, Cat # A-11078, dil. 1 : 200) for 1 hour. All the samples were then washed three times in PBS and mounted with VECTASHIELD® antifade mounting medium with DAPI for nuclear staining (Vector Laboratories, Cat. # H-1200).

### 2.6. Detection of MION Labeled Isl-1^+^ Cells (Prussian Blue Staining)

The excised hearts were embedded in paraffin and sliced into 4 *μ*m thick sections for histological analysis. After deparaffinization with xylene, samples were rehydrated through an ethanol gradient and rinsed in distilled water. After the rehydration steps, some sections of each heart were stained with Prussian Blue to examine the biodistribution of MION-labeled CSC. Briefly, tissue sections were washed 3 times with PBS, incubated for 30 minutes with 5% potassium ferrocyanide (Sigma-Aldrich, St. Louis, MO, USA) in 5% hydrochloric acid, and rewashed and counterstained with nuclear fast red (Sigma) for 20 minutes.

### 2.7. Western Blot Studies

Isolated CPC were lysed with RIPA lysis buffer (Sigma-Aldrich, St. Louis, MO), and cell suspension was centrifuged at 15,000 g to pellet cell debris. Protein concentration was measured in the supernate using the BCA Protein Assay Reagent (Pierce, Rockford, IL, USA) at an absorbance of 562 nm as per the manufacturer's directions.

Electrophoresis was performed using a discontinuous Tris-glycine buffer system. Proteins were suspended in sodium dodecyl sulfate polyacrylamide gel electrophoresis (SDS-PAGE) buffer (20 mM Tris-HCl, 0.2 M glycine) and boiled for 5 min. Forty micrograms of protein was loaded in each well of a 10% SDS polyacrylamide gel. Gels were immediately electrotransferred to polyvinylidene difluoride (PVDF) membranes (Millipore, Bedford, MA, USA) at 60 V for 1 h by means of a semidry transfer system (Bio-Rad Lab., Hercules, CA, USA) (transfer buffer: 20 mM Tris-HCl, 0.2 M glycine, 20% MeOH). Membranes were blocked with 5% nonfat dry milk in TBS/Tween buffer (blocking buffer, BB) (25 mM Tris-HCl, 0.14 M NaCl, 2% Tween20; Bio-Rad Lab) for 1 hour at room temperature. Membranes were then cut in strips, and each lane was incubated with either rabbit anti-ISL-1 (Aviva Systems Biology, San Diego, CA) dil. 1 : 1000, goat anti-CSX1/NKX2-5 (Everest Biotech Ltd., Upper Heyford Oxfordshire, UK), mouse anti-GATA4 (Abcam Inc., Cambridge, MA) dil 1 : 500, rabbit anti-c-kit (Novus Biologicals, Littleton, CO) dil 1 : 500, rabbit anti-sarcomeric Alpha Actinin (Abcam) dil 1 : 500, mouse anti-cardiac Troponin I (Abcam) dil. 1 : 500, or rabbit anti-Connexin 43 (Invitrogen, Camarillo, CA) (4 *μ*g/ml) in blocking buffer. Membranes were then washed three times, 5 minutes each time, with TBS containing 0.05% Tween-20 followed by incubation with one of the following HRP-conjugated secondary antibodies: goat anti-rabbit (1 : 200), goat anti-mouse (1 : 200), and donkey anti-goat (1 : 200) (Santa Cruz Biotechnology, Santa Cruz, CA) at room temperature for 1 hour. Following three washes as above, proteins were revealed by enhanced chemioluminescence (ECL) (Amersham, Buckinghamshire, England) were exposed to film (Hyperfilm ECL; Amersham). The film was scanned, and protein band concentrations were quantified by integrated optical density using NIH ImageJ software (NIH).

### 2.8. Detection and Quantification of Fibrotic Tissue

Interstitial fibrosis was assessed on 5 *μ*m thick paraffin embedded tissue sections by Masson's trichrome staining (Accustain™ Trichrome (Masson), kit # HT-15, Sigma-Aldrich, St. Louis, MO.) as per the manufacturer's instructions. Stained glass slides were optically scanned using a ScanScope XT digital slide scanner (Aperio Technologies, Inc., Vista, CA) to create whole-slide digital planar image files at 0.5 *μ*m/pixel resolution. Quantification of fibrotic areas was achieved using Aperio ImageScope software. The standard positive pixel algorithm was altered to read the Masson's Trichrome stained slides (hue value: 0.62; hue width 0.4; color saturation threshold: 0.005). The total area was then selected by tracing the outer margin of the tissue section using the pen tool while the void areas (right and left ventricles) were subtracted by tracing them with the negative pen tool. After the analysis was run, the markup image, showing the contractile tissue in blue and the fibrosis in red, was generated, and the resulting values were displayed in the annotations window and appeared as positivity = Npositive/Ntotal. Values were then multiplied by 100 to calculate the percentage of total area. Values were then expressed as mean ± SEM. Wall thickness was measured also with ImageScope using the ruler tool. In each tissue section, seven different measurements were taken across the infarcted area (left ventricle anterior wall) and averaged.

### 2.9. Y-Chromosome Fluorescence In Situ Hybridization

Five-micrometer sections of PFA-fixed, paraffin-embedded cardiac tissue were deparaffinized in xylene, rehydrated through a graded ethanol series, postfixed in 10% neutral-buffered formalin (NBF) for 10 minutes, and rinsed in water before air drying for one hour. Slides were pretreated in 0.2 N HCl for 30 minutes at room temperature and then washed twice for 5 minutes in ddH_2_O. Antigen retrieval was performed by immersing slides in 1 M sodium thiocyanate for 30 minutes at 85°C. The slides were removed from the retrieval solution and rinsed thoroughly in ddH_2_O. Sections were digested in 4 mg/ml pepsin (Sigma) diluted in 0.9% NaCl (pH 2.0) for 55 minutes at 37°C. Digested sections were removed and immediately rinsed in water, equilibrated in 2× saline-sodium citrate (SSC), and dehydrated through a graded ethanol series. Ten microliters of rat Y + chromosome 12 probe (Cambio Ltd.; Cambridge, UK) was denatured for 10 minutes in a 75°C water bath and allowed to preanneal at 37°C for 45 minutes prior to being applied to the prepared section and topped with a 22 × 22 coverslip. Slides were sealed with rubber cement, denatured at 75°C for 6 minutes, and hybridized overnight at 37°C in a Hybrite oven (Vysis, Downers Grove, IL). After hybridization, coverslips were removed and sections were washed in 3 changes of 50% formamide/2 × SSC, followed by 2 × SSC, and 4 × SSC + 0.1% Igepal (NP-40) at 46°C for 7 minutes each. Slides were air dried at RT in the dark and mounted with Vectashield® mounting medium with DAPI (Vector Laboratories).

### 2.10. Immunostaining for von Willebrand Factor

All steps were performed using DAKO Autostainer Plus System (Agilent, Santa Clara, CA). Five-micrometer sections of PFA-fixed, paraffin-embedded cardiac tissue were processed for immunohistochemistry using DAKO PT Link (Agilent). Briefly, samples were deparaffinized in xylene and rehydrated through a graded ethanol series. Antigen retrieval was achieved using EnVision FLEX high pH target retrieval solution (Agilent). The cycle begins at 85°C and is heated to 100°C for 20 minutes and then cooled back to 85°C. The slides were then removed from the retrieval solution and rinsed thoroughly in Dako wash buffer. Samples were then incubated for 10 min in H_2_O_2_ (Thermo Fisher Scientific Waltham, MA), rinsed in Dako wash buffer (Agilent), and incubated for 15 minutes in serum-free Dako protein block to inhibit nonspecific staining. von Willebrand factor antibodies (Cat. # IR 527 TRS; DAKO-Agilent) were applied for 20 minutes at room temperature, and after a quick rinse with wash buffer, samples were incubated with EnVision FLEX HRP (Cat. # K8000; Dako-Agilent) for 10 minutes. After another rinse in wash buffer, samples were incubated for 10 minutes in diaminobenzidine (DAB) followed by a quick rinse and two baths of 5 minutes in hematoxylin. Samples were then rinsed in DI H_2_O and dehydrated through a graded ethanol series and mounted.

### 2.11. Animal Studies

All protocols conformed to the National Institutes of Health (NIH) guidelines. Animals received care in compliance with the Principles of Laboratory Animal Care. All rats were housed with access to food and water *ad libitum* on a 12 h/12 h light/dark cycle.

### 2.12. LAD Ligation

Six-week-old Sprague-Dawley rats (Charles River Laboratories, Inc., Wilmington, MA) were initially anesthetized with 5% isoflurane. Endotracheal intubation was then performed by insertion of an endotracheal tube (polyethylene size 90; PE 90), and mechanical ventilation was then achieved by connecting the endotracheal tube to a Harvard Model 683 Small Animal Ventilator (Harvard Apparatus, Holliston, MA).

Throughout the duration of the surgery, rats were exposed to a 3% isoflurane flow.

After the LAD coronary artery was exposed through a 4th intercostal left lateral thoracotomy, the pericardium was cut open. The LAD coronary artery was ligated with a 5-0 silk suture at 1-2 mm from the left auricle, with slight excursion of the cross of the left auricle and right conus branch. A successful MI was established upon sudden discoloration of the anterior wall of the left ventricle.

**Figure 1:**
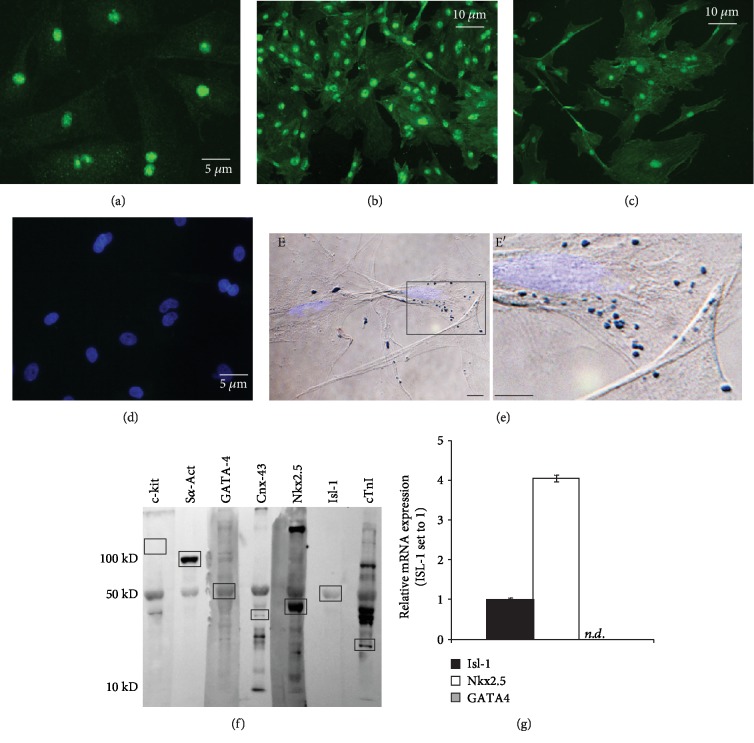
Characterization of isolated embryonic cardiac stem cells in culture. (a)–(c) Immunofluorescence micrographs showing cells isolated from E12.5 rat hearts expressing various cardiac progenitor markers: Isl-1 (a), Nkx2.5 (b), and GATA-4 (c). (d) Blank, in which the primary antibody was omitted. (e) Bright field and superimposed fluorescence micrograph of cells stained with Prussian Blue demonstrate incorporation of MION in the cytoplasm A close-up view of (e) is shown (e'). Light blue: nuclei (DAPI); dark blue: MION. (f) Western blot analysis of cell extract confirmed expression of various cardiac markers (Islet-1, Nkx2.5, GATA-4, sarcomeric *α*-actinin, cardiac troponin I, connexin-43). Cells were identified as c-kit^−^.(g) Real-time PCR analysis showed that, after several passages in culture, isolated cells lost expression of GATA-4.

After LAD ligation, rats were randomly assigned to one of the following groups and received intramyocardial injection (100 *μ*l) of either 0.9% NaCl solution (*n* = 13) (saline), *in vitro*-expanded rat fetal Isl-1^+^ cells (1 × 10^6^) (*n* = 12) (Isl-1), freshly isolated CD90^+^ cells (1 × 10^6^) (*n* = 10) (CD90), or a combination of Isl-1^+^ and CD90^+^ cells(Isl-1/CD90) (1 × 10^6^/ea.) (*n* = 9), at time of infarction delivered transepicardially to the peri-infarct zone. An aliquot of Isl-1+ cells was plated for purity assessment by immunohistochemistry and showed a near to 100% positivity for Isl-1 (Figure 1(a)). A fifth group (sham) (*n* = 5) was composed by age/sex-matched rats which did not receive any surgery or injections. All rats were euthanized 4-week post-MI with pentobarbital (50 mg/kg). Cardiac tissue was quickly harvested and processed for histology.

### 2.13. Echocardiography

Rats were lightly anesthetized with isoflurane (2%) O_2_ mixture via a face mask attached to a gas vaporizer during spontaneous ventilation. Animals were imaged in a shallow left lateral supine position using a GE vivid7 ultrasound machine with a 12 MHz transducer (GE Healthcare, NJ, USA) to assess the ventricular dimensions and cardiac function. ECG electrodes were placed for timing of intracardiac events. In short-axis view, using 2D and M mode images, posterior wall thickness, end-systolic and end-diastolic dimensions, and % fractional shortening were measured and averaged from 3 cardiac cycles. 2D guided pulsed Doppler recordings of mitral inflow were obtained in the apical 4-chamber view with the sample volume placed at the tips of the mitral leaflets in diastole. Peak flow velocities of early filling (*E*) and late filling (*A*), *E*/*A* ratio, rate of decrease in early flow (*E*_dec_slope), and deceleration time (*E*_dec_time) were averaged from 6 consecutive cycles. Load-independent spectral tissue Doppler measurements from the septal mitral annular wall were assessed for *E*′ (myocardial elongation during early filling), *A*′ (myocardial wall motion during late filling), *E*′/*A*′, and *E*/*E*′ (estimation of LV filling pressures). All echocardiographic studies were performed and analyzed by an investigator/echocardiographer that was masked to the treatment groups.

Imaging required 10 minutes per rat. Respiratory rate (e.g., adequate diaphragm excursion) was followed by clinical observation throughout the scanning period. Prior to anesthesia induction, all animals were weighed.

### 2.14. Direct Hemodynamic Evaluation

Four weeks after MI induction, rats were anaesthetized initially with a 5% isoflurane/O_2_ mixture in an induction box and then kept asleep with a 2.5% isoflurane/O_2_ mixture via a face mask attached to a gas vaporizer during spontaneous ventilation. Rats were placed in the supine position, and body temperature was maintained at 37°C by a heating pad throughout the experiment. Left ventricular function was measured using a pressure catheter (SPR-320; Millar Instruments, Houston, TX, USA), which was inserted into the right carotid artery. After 10 min stabilization, arterial blood pressure was recorded for 10 min. Then, the catheter was advanced into the left ventricle and stabilized for 5 min. The waveform was used to confirm the positioning of catheter in the LV. Left ventricular end-diastolic pressure (LVEDP) was continuously recorded for 10 min by the Powerlab Chart 4 software system (ADInstruments Inc., Colorado Springs, CO, USA).

### 2.15. Statistical Analysis

All experiments were repeated at least three times. The hemodynamic evaluation was assessed using a Student's *t*-test plus ANOVA for multiple comparisons. Results are expressed as mean ± SEM. Statistical analysis was performed using EXCEL software (Microsoft Corp., Redmond, WA) with *P* < 0.05 considered statistically significant. All the examiners were blinded to the identity of the samples they were analysing.

## 3. Results and Discussion

We isolated embryonic cardiac-derived cells and demonstrated the expression of cardiac precursor markers: Isl-1, Nkx2.5, and GATA4 (Figures 1(a)–1(d)) and their ability to incorporate MION as demonstrated by Prussian Blue staining (Figures 1(e) and 1(e')). Near to 100% of the cells stained were ISL-1^+^. MION incorporation would facilitate in vivo tracking of these cells. Analysis of whole cell extract using Western blot confirmed the expression of the abovementioned markers as well as the cardiac markers, sarcomeric *α*-actinin, cardiac troponin I Western and the gap junction protein connexin-43 (Figure 1(f)); the latter protein has been deemed critical for the conduction of cardiac action potential. Cells were also confirmed to be c-Kit^−^ (Figure 1(f)). Adult Isl-1^+^ cardiac progenitor/stem cells can be c-Kit^+^ [28] or c-Kit^−^ cells [11]. Simpson et al. have reported that neonatal-derived Isl-1^+^c-Kit^+^ cells have greater regenerative ability than their adult counterparts [29]. However, few studies have utilized embryonic Isl-1^+^ cardiac progenitors either c-Kit^+^ [30, 31] or c-Kit^−^ [32] as we did in this study. Expression of Isl-1, Nkx2.5, and GATA4 was preserved throughout the study, except that continued passage resulted in loss of GATA4 expression as demonstrated by real-time PCR (Figure 1(g)).

We also utilized the bone marrow stem cell population, CD90^+^ cells. CD90 or Thy-1 is a 25–37 kDa glycosylphosphatidylinositol- (GPI-) anchored glycoprotein and is highly expressed in all MSCs, irrespective of the source, and it is a good marker for CFU-F enrichment [33]. High CD90 expression has also been related to the undifferentiated status of MSCs, since a decrease in CD90 level can be correlated with the temporal lineage commitment in vitro [34].

**Figure 2:**
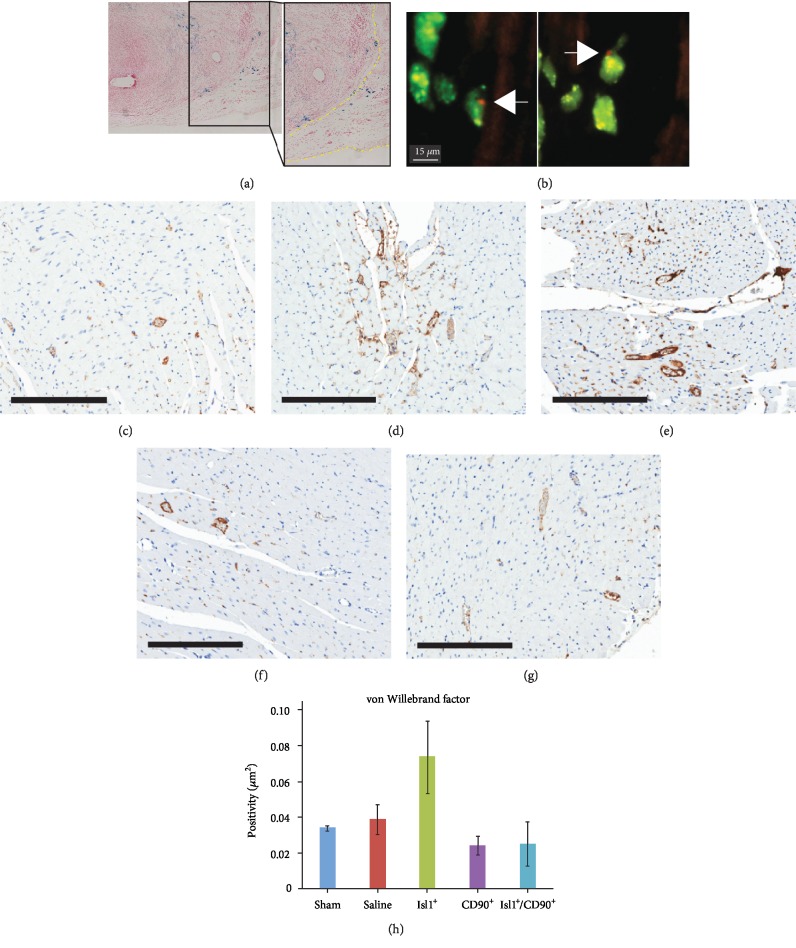
Biodistribution of injected cells using MION labeling and FISH, showing that cell injections do not increase vascular density: (a) Isl-1^+^ cells retain MION labeling, as shown by Prussian blue staining, 4 weeks following injection with cells infiltrating the scar tissue (between yellow dotted lines) suggesting participation in the repair process. (b) Male donor-derived CD90^+^ cells were detected 4-week post-MI by fluorescence *in situ* hybridization (FISH) for the Y chromosome shown in red (arrows). (c)–(g) Representative images from all cohorts of cardiac tissue sections stained for von Willebrand factor. (c) sham; (d) saline; (e) Isl-1^+^; (f) CD90^+^; (g) Isl-1^+^/CD90^+^). Scale bar: 200 *μ*m. (h) Quantification of all the sections analyzed (saline: 0.0388 *μ*m^2^ ± 0.008; sham: 0.0340 *μ*m^2^ ± 0.002; Isl-1: 0.0737 *μ*m^2^ ± 0.02; CD90^+^: 0.02 *μ*m^2^ ± 0.005; Isl-1^+^/CD90^+^: 0.025 *μ*m^2^ ± 0.01).

To evaluate the reparative potential of Isl-1^+^cells alone or in combination with CD90^+^ cells, we injected cells in the peri-infart region of rats undergoing the LAD ligation model of MI. The biodistribution of the cells was examined by MION labeling of Isl-1^+^ cells and fluorescence *in situ* hybridization (FISH) staining of donor-derived CD90^+^ cells. Prussian Blue staining of paraffin embedded sections showed that Isl-1^+^ cells retained MION labeling at 4-week post-MI and that the Isl-1^+^cells infiltrated the fibrous tissue suggesting their participation in the repair process (Figure 2(a)). This would suggest that the MION-labelled cells migrated into the infarcted region and remained there. We did not observe any evidence of chroninc inflammation in the peri-infarct or infarct area to suggest that the MION was simply taken up by infiltrating macrophages, but rather our results would suggest that the MION was retained within the Isl-1^+^ cells. Previous studies have also reported the retention of MION particles into transplanted cells up to 28 days of posttransplantation [35] Donor-derived CD90^+^ cells were detected in and around the infarct region 4-week post-MI. FISH for the Y chromosome (Figure 2(b), arrows) demonstrated that CD90^+^cells home to the site of injury and were found 4-week postinjection. The presence of FISH for the Y chromosome in the 4-week samples would suggest that the detected cells were still viable at this time point.

A critical repair response following MI is restoration of the microvasculature in the injured area. To determine whether administration of Isl-1^+^ and/or CD90^+^cells increased capillary density in the peri-infarct region, von Willebrand factor staining was performed on serial cross sections of the heart. Unexpectedly, there was no significant increase in any of the groups when compared to saline-injected controls (saline: 0.0388 *μ*m^2^ ± 0.008 vs. sham: 0.0340 *μ*m^2^ ± 0.002; *p* = 0.36; saline vs. Isl-1: 0.0737 *μ*m^2^ ± 0.02; *p* = 0.06; saline vs. CD90^+^: 0.02 *μ*m^2^ ± 0.005; *p* = 0.09; saline vs. Isl-1^+^/CD90^+^: 0.025 *μ*m^2^ ± 0.01; *p* = 0.21). (Figures 2(c)–2(h)). This may be because our assessment was not sensitive enough to detect a change as we only made measurement in two dimentions and the capillary network is an elaborate 3-dimentional structure.

**Figure 3:**
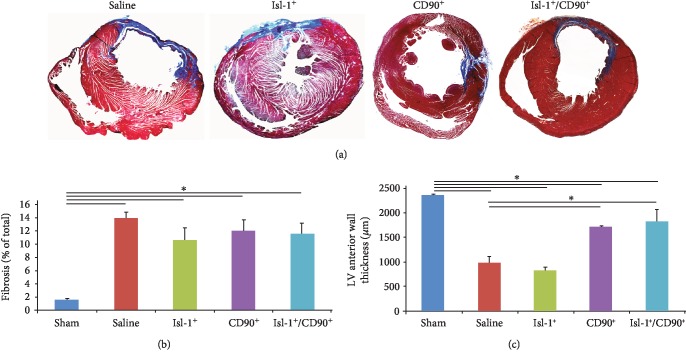
Combination of Isl-1^+^ cells/CD90^+^ cells preserves wall thickness. (a) Masson's trichrome staining was used to stain the fibrotic tissue in blue. Representative bright field microscopy images of 4-week post-MI rat hearts are shown. Fibrosis is limited to the anterior wall of the left ventricle. (b) Quantification of fibrosis revealed that none of the cell treatments was able to significantly reduce fibrosis (saline: 13.85% ± 0.96 vs. Isl-1^+^: 10.69% ± 1.77, *p* = 0.14; saline vs. CD90^+^: 11.95% ± 1.61, *p* = 0.22; saline vs. Isl-1^+^/CD90^+^: 11.62% ± 1.55, *p* = 0.19). The combination of Isl-1^+^cells and CD90^+^ cell treatment was sufficient to maintain wall thickness (sham: 2354.39 *μ*m ± 18.03 vs. saline: 996.98 *μ*m ± 120.41, *p* < 0.05; sham vs. Isl-1^+^: 834.91 *μ*m ± 54.50, *p* < 0.05; sham vs. CD90^+^: 1711.40 *μ*m ± 16.67, *p* < 0.05; sham vs. Isl-1^+^/CD90^+^: 1841.23 *μ*m ± 228.08, *p* = 0.02) (c).

Masson's trichrome staining of 4-week post-MI hearts (Figure 3(a)) revealed that fibrosis was, as expected, limited to the anterior wall of the left ventricle. Surprizingly, quantification of the fibrosis demonstrated that no cell treatment was able to significantly reduce fibrosis when compared to saline-treated rats (saline: 13.85% ± 0.96 vs. Isl-1^+^: 10.69% ± 1.77, *p* = 0.14; saline vs. CD90^+^: 11.95% ± 1.61, *p* = 0.22; saline vs. Isl-1^+^/CD90^+^: 11.62% ± 1.55, *p* = 0.19) (Figure 3(b)). In contrast, the Isl-1^+^/CD90^+^ combination was capable of preserving wall thickness (sham: 2354.39 *μ*m ± 18.03 vs. saline: 996.98 *μ*m ± 120.41, *p* < 0.05; sham vs. Isl-1^+^: 834.91 *μ*m ± 54.50, *p* < 0.05; sham vs. CD90^+^: 1711.40 *μ*m ± 16.67, *p* < 0.05; sham vs. Isl-1^+^/CD90^+^: 1841.23 *μ*m ± 228.08, *p* = 0.02) (Figure 3(c)).

**Figure 4:**
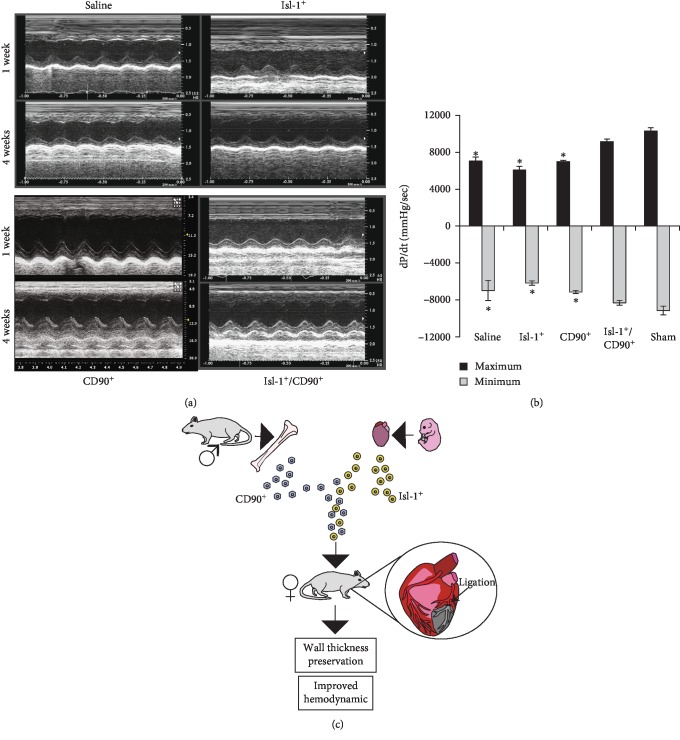
Combination of Isl-1^+^cells and CD90^+^ cells leads to improved cardiac function, and schematic representation of combination stem cell administration on cardiac repair following LAD ligation induced MI. (a) Representative screenshots of echocardiograms at 1- and 4-week post-MI time points in all the four treatment groups (saline, Isl-1^+^, CD90^+^, and Isl-1^+^/CD90^+^). (b) Hemodynamic parameter assessment by Millar catheterization revealed that the combination therapy group demonstrated a significant improvement of cardiac function compared to either monotherapy. There was no significant difference between Isl-1^+^/CD90^+^ and sham. (c) Isl-1^+^ cells derived from mouse embryos and adult bone marrow-derived CD-90^+^ cells work in combination to restore cardiac function and preserve wall thickness in the LAD ligation model of MI. In vivo cells work in synergy to carry out healing and the use of two cell types may serve to optimize outcomes both for the target tissue in need of repair and for facilitating donor cell survival.

Echocardiography was used to monitor the extent of the infarct at one and four weeks of post-MI (Figure 4(a)). Analysis of the recorded short-axis 2D and M mode images from the same rats at 1- and 4-week post-MI revealed no improvement of the anterior wall contractility in the saline-treated rats. Modest improvement of wall motion was observed in the Isl-1^+^ cell-treated rats and in CD90^+^ cell-injected rats. However, a complete recovery of wall contractility was observed in the rats receiving combination of Isl-1^+^/CD90^+^ cells. The rate of LV pressure, dP/dt (max), a measure of ventricular systolic contractility, improved only in the animals treated with the Isl-1^+^/CD90^+^cell combination, restoring cardiac function back to physiologic levels (Isl-1^+^/CD90^+^: 8982 mmHg/sec ± 270 vs. sham: 10,111 mmHg/sec ± 339; *p* = 0.159; saline: 6910 mmHg/sec ± 439 vs. sham; *p* < 0.05; Isl-1^+^: 5971 mmHg/sec ± 368 vs. sham; *p* < 0.05; CD90^+^: 6865 mmHg/sec ± 130 vs. sham; *p* < 0.05) (Figure 4(b)). Furthermore, dP/dt (min), a measure of ventricular diastolic relaxation, showed complete restoration of cardiac function in the combination group (Isl-1^+^/CD90^+^: −8169 mmHg/sec ± 251 vs. sham: −8992 mmHg/sec ± 464; *p* = 0.223; saline: −6910 mmHg/sec ± 1057 vs. sham; *p* = 0.057; Isl-1^+^: −6070 mmHg/sec ± 222 vs. sham; *p* < 0.05; CD90^+^: −7028 mmHg/sec ± 155 vs. sham; *p* < 0.05). Taken together, administration of the Isl-1^+^/CD90^+^ cell combination following MI improved cardiac contractility to the greatest degree and resulted in improved functional outcomes compared to either cell type alone.

One limitation of this report is that it represents a single-dose study; therefore, the observed lack of benefit of either Isl-1^+^ or CD90^+^ type alone could be due to insufficient numbers. Furthermore, the beneficial effect we observed in the combination cell experiment may be due to the injection of two million cells instead of one million. Future studies are needed to test the efficacy of multiple doses and different proportions of each test population per dose. With repeated injections of the individual populations, efficacy in the endpoints examined may occur. Eventhough CD90^+^cells alone did not provide functional benefit, the cells were detectable four weeks post-MI suggesting that their prolonged survival offered paracrine support to the Isl-1^+^ cells, in turn, fostering their survival and function. The Isl-1^+^ cells when injected expressed Nkx2.5, GATA4, *α*-sarcomeric actin, cardiac troponin-I, and connexin 43 and represented immature cardiomyocytes that were progressing towards linage specification; however, we did not determine that the cells actually differentiated into cardiomyocytes *in vivo* and the beneficial effect we observed may likely be due to the paracrine production of these cells. Future studies may consider the use of Isl-1^+^ cells with different levels of differentiation and the use of fluorescently labeled Isl-1^+^ cells to facilitate tracking and colocalization with cardiomyocyte specific markers to establish whether the Isl-1^+^cells can differentiate *in vivo* to cardiomyocytes.

With the wide spread utilization of iPSC technology and now initiation of clinical trials utilizing these cells, the possibility of generating large numbers of cells with fetal phenotypes and surface markers is now possible. Moretti et al. generated Isl1 cells from iPSC making the transition to generation of patient specific Isl-1^+^ cells feasible [36]. Furthermore, the use of SSEA5^−^ iPSCs reduces the risk of teratomas making the transition of iPSC technology to clinical trials more achievable [37].

## 4. Conclusions

In summary, Isl-1^+^/CD90^+^ cell combination restored cardiac function and preserved wall thickness in the rat MI model and provides support for the consideration of combinations of cells for therapeutic interventions. In vivo, cells work in synergy to carry out healing and the use of two cell types may serve to optimize outcomes both for the target tissue in need of repair and for facilitating donor cell survival. Determining which cell combinations function optimally for a particular disease or injury model will represent a new challenge.

## Data Availability

The data used to support the findings of this study are included within the article.
